# Association Between Wearable Device Use and Quality of Life in Patients With Idiopathic Inflammatory Myopathies and Primary Systemic Vasculitis

**DOI:** 10.7759/cureus.58948

**Published:** 2024-04-24

**Authors:** Nathalia G Pereira, Alexandre M Dos Santos, Samuel K Shinjo

**Affiliations:** 1 Division of Rheumatology, Faculdade de Medicina da Universidade de São Paulo (FMUSP), Sao Paulo, BRA

**Keywords:** systemic vasculitis, systemic autoimmune myopathies, idiopathic inflammatory myopathies, wearable devices, vasculitis, physical activity, myositis, quality of life, inflammatory myopathies, fatigue

## Abstract

Background

Despite the increasing use of wearable devices worldwide, concise data on these instruments in patients with systemic autoimmune rheumatic diseases, including idiopathic inflammatory myopathies (IIM) and primary systemic vasculitis (PSV), are lacking.

Objectives

The aim of this study is to investigate the knowledge and use of wearable devices and to assess their impact on the general quality of life of patients with IIM and PSV. Moreover, we compared these characteristics between patients with IIM and PSV users and non-users of wearable devices.

Methods

This single-center, cross-sectional study was conducted between January 2023 and June 2023. We included adult patients with IIM and PSV and a control group (CTR) and evaluated their use of cell phones and wearables, level of physical activity, and quality of life.

Results

A total of 132 patients with IIM, 82 with PSV, and 178 in the CTR were evaluated. Overall, 169 patients and 144 in the CTR were aware of wearable devices, of whom 50 (29.6%) and 47 (32.6%), respectively, had already used this technology. In addition, the IPAQ-Mets and EQ-5D scores were lower in the IIM and PSV groups than in the CTR, and the fatigue severity scale (FSS) scores were higher in the IIM and PSV groups than in the CTR. Patients who used the devices showed FSS scores of 29 (18-40) points, with higher levels of IPAQ-Mets among device users, indicating greater physical activity than among nonusers.

Conclusion

Based on the results, the use of wearable devices is associated with better fatigue and IPAQ scores. Possibly, the use of such devices can have an impact on better lifestyle habits among these patients.

## Introduction

Idiopathic inflammatory myopathies (IIM) or systemic autoimmune myopathies are rare muscular diseases characterized by the clinical presentation of skeletal muscle weakness. Furthermore, IIM may be accompanied by extramuscular manifestations such as cutaneous, pulmonary, cardiac, articular, and gastrointestinal involvement, thereby diminishing the patient’s quality of life and functional capacity [1-3]. Dermatomyositis, antisynthetase syndrome, polymyositis, inclusion-body myositis, and immune-mediated necrotizing myopathies are part of IIM.

Primary systemic vasculitis (PSV) is a heterogeneous group of rare diseases that exhibits variability in clinical, radiological, and laboratory presentations. These conditions are characterized by the dysregulation of immune modulation, revealing an inflammatory process primarily in the blood vessels associated with damage to target organs [4]. Takayasu arteritis, giant cell arteritis, polyarteritis nodosa, granulomatosis with polyangiitis, eosinophilic granulomatosis with polyangiitis, and cryoglobulinemic vasculitis are part of PSV.

As a global trend, telemonitoring using wearable devices to compile clinical data and lifestyle habits has been employed as a supplementary strategy [5,6]. One wearable device that has gained prominence is designed to monitor physical activity levels. These devices encompass high-complexity options (e.g., watches and bracelets integrated with clinical data) and low-complexity options (e.g., smartphone applications) [7,8].

This monitoring process has become automated owing to the wearable technology. These devices can be synchronized with computer applications on multiple devices such as smartphones. By transferring data to these devices, users can review their physical activity history and use this information to make behavioral adjustments to their physical activity [9]. Moreover, wearables can help patients actively participate in their health care and potentially improve their quality of life, particularly in chronic disease management and elderly care. Pardamean et al. [7] demonstrated that technology can instigate behavioral changes and potentially motivate engagement in physical activities.

However, despite the growing use of these instruments as complementary tools, there is still a lack of concise data on patients with systemic autoimmune rheumatic diseases including IIM and PSV. It is crucial to investigate the knowledge and utilization of low-complexity physical activity devices, considering their impact on quality of life and patient-centered outcomes, for an improved clinical approach and non-pharmacological strategy. Therefore, the aims of the present study were: (a) to investigate the knowledge and use of wearable devices and to assess their impact on the fatigue, physical activity level, fatigue scores, and general quality of life of patients with IIM and PSV, and (b) to compare these characteristics between patients with IIM and PSV users and non-users of wearable devices.

## Materials and methods

This single-center cross-sectional study was conducted in our tertiary center between January 2023 and June 2023. The study was approved by the local ethics committee (CAAE 55715522.0.0000.0068). All the participants signed an informed consent form and were provided with instructions regarding the purpose of the study.

We included adult patients with IIM diagnosed with dermatomyositis, polymyositis, inclusion-body myositis, and immune-mediated necrotizing myopathies, according to the classification criteria of EULAR/ACR 2017 [10]; those diagnosed with antisynthetase syndrome, according to Behrens Pinto et al. [11]; and adult patients with PSV diagnosed with Takayasu arteritis, giant cell arteritis, polyarteritis nodosa, granulomatosis with polyangiitis, eosinophilic granulomatosis with polyangiitis, and cryoglobulinemic vasculitis [12,13], who underwent regular outpatient follow-ups at our tertiary center. Concurrently, individuals without rheumatic diseases were screened and recruited as the control group (CTR) online through a form and explanatory video about wearable devices and the research objectives, and in person, involving volunteers from our tertiary center who do not have rheumatic diseases.

Data collection

An online and public e-survey was developed using the REDCap© data management tool version 11.2.5. The questionnaire was pilot-tested with experienced health care professionals to assess grammatical errors, validity, and representativeness. Subsequently, pilot tests were conducted on patients with IIM and PSV and those in the CTR to validate the constructs of the questions. The e-survey was applied with the assistance of an interviewer (one of the authors of the present study).

The following parameters were collected from patients through the survey: demographic, socioeconomic, and educational data; current age; ethnicity; education level; use of mobile phones and wearable devices; type and duration of disease; comorbidities (e.g., systemic arterial hypertension, dyslipidemia, and diabetes mellitus); level of physical activity (International Physical Activity Questionnaire - Short Form) [14] (range: 0 to 100); visual analog scale for fatigue (VASf) [15] (range: 0 [no fatigue] to 10 [severe fatigue]); VAS for pain (VASd) [16] (range: 0 [no pain] to 10 [severe pain]); state of health (EQ-5D) score [17] (a standardized measure of health-related quality: no problems = 1; some problems = 2; and extreme problems or unable to = 3, in five items: mobility, self-care, usual activities, pain/discomfort, and anxiety/depression); and fatigue severity scale (FSS) [18] (nine items about fatigue, its severity and how it affects certain activities. Answers are scored on a seven-point scale, where 1 means strongly disagree and 7 means strongly agree; therefore, the minimum score possible is 9 and the highest is 63. The higher the score, the more severe the fatigue.

Statistical analysis

Data were presented as median (25th-75th), relative frequency (%), and/or absolute frequency (n). Parametric quantitative variables were compared using the t-test and its T-statistic for two groups or using the analysis of variance (ANOVA) and its F-statistic for three groups, whereas non-parametric and ordinal qualitative variables were assessed using the Mann-Whitney U test and its W-statistic or the Kruskal-Wallis test and its chi-squared statistic. The chi-squared test or Fisher’s exact test was used for categorical variables. For post hoc tests to compare differences between groups, we employed the Tukey test for ANOVA, the Nemenyi test for Kruskal-Wallis, and individual chi-squared tests for proportions within each group. We considered the significance level at P < 0.05. All statistical analyses were performed using R Version 4.3.2 (R Foundation for Statistical Computing, Vienna, Austria).

The sample size was determined based on the need to detect a difference among the medians of the three groups, with specific reference to the ANOVA test, representing the comparison of our primary outcome. An effect size of 0.25 was employed, with a significance level (α) of <0.05, a power (1 - β) of 0.95, and allocation across the three groups. Consequently, a sample of 249 individuals was deemed necessary and evenly distributed among the three groups (IIM, PSV, and CTR). Sample size calculations were performed using g* power v.3.1.9.6 (Windows, University of Kiel, Kiel, Germany).

## Results

A total of 132 patients with IIM, 82 with PSV, and 178 CTR were assessed. Among the patients with IIM, 65 (49.2%) had dermatomyositis, 35 (26.5%) had antisynthetase syndrome, 18 (13.7%) had polymyositis, 13 (9.8%) had necrotizing immune-mediated myopathy, and one (0.8%) had inclusion body myositis. Among patients with PSV, 38 (46.3%) had Takayasu arteritis, 24 (29.3%) had granulomatosis with polyangiitis, 13 (15.9%) had polyarteritis nodosa, three (3.7%) had eosinophilic granulomatosis with polyangiitis, two (2.4%) had cryoglobulinemic vasculitis, and two (2.4%) had giant cell arteritis.

The current mean age and distribution of White ethnicity, BMI, marital status, and high school level were comparable between the IIM group vs CTR and the PSV group vs CTR. However, the frequency of the female sex distribution was higher in the IIM and PSV groups than in the CTR (Table 1). In addition, disease duration was longer in the PSV group than in the IIM group.

**Table 1:** Demographic features and disease duration of patients with IIM and PSV, and the CTR Data are presented as the median (25th-75th) or number (%) *F-statistic; **Tukey's test statistic (post-hoc); †W-statistic; ††Kruskal-Wallis chi-squared statistic; †††Nemenyi test statistic; ‡chi-squared statistic CTR, control group; BMI, body mass index; IIM, idiopathic inflammatory myopathies; PSV, primary systemic vasculitis

	IIM (n=132)	PSV (n=82)	CTR (n=178)	P-value	Statistic of test	P-value (post hoc)			Statistic of test (post hoc)		
						IIM vs. CTR	PSV vs. CTR	IIM vs. PSV	IIM vs. CTR	PSV vs. CTR	IIM vs. PSV
Age (years)	48 (37.7-58.0)	45 (36.2-54.7)	48 (38.0-58.0)	0.608	0.498*	0.693	0.681	0.991	-1.153**	-1.180**	-0.178**
White ethnicity	42 (31.8)	37 (45.1)	88 (49.5)	0.007	9.892‡	0.002	0.607	0.070	8.954‡	0.264‡	3.294‡
Female	95 (71.9)	57 (69.5)	149 (83.7)	0.011	8.936‡	0.018	0.013	0.817	5.551‡	6.039‡	0.053‡
BMI (kg/m²)	28.8 (23.6-33.6)	27.3 (24.3-30.2)	27.3 (24.2-31.2)	0.190	3.303††	0.406	0.745	0.190	1.812†††	1.033†††	2.460†††
Marital status (married)	52 (39.4)	35 (42.7)	91 (51.2)	0.104	4.517‡	0.053	0.257	0.739	3.737‡	1.281‡	0.111‡
High school degree	45 (34.1)	33 (40.2)	77 (43.3)	0.261	2.686‡	0.129	0.747	0.445	2.299‡	0.104‡	0.582‡
Disease duration (years)	7 (4.0-11.0)	12 (5.2-17.7)	-	<0.001	3696.5†	-	-	-	-	-	-

The frequency of diabetes, dyslipidemia, and level (low or high) of physical activity were also compared between the groups. However, the prevalence of systemic arterial hypertension was higher in the PSV group than in the CTR, and the IPAQ-Mets score was lower in the IIM and PSV groups than in the CTR (Table 2).

**Table 2:** Chronic fatigue, comorbidities, quality of life, and level of activities of daily living of patients with IIM and PSV, and the CTR Data are presented as the median (25th-75th) or number (%) ††Kruskal-Wallis chi-squared statistic; †††Nemenyi test statistic; ‡chi-squared statistic CTR, control group; EQ-5D, quality of life questionnaire; FSS, fatigue severity scale; IIM, idiopathic inflammatory myopathies; IPAQ, International Physical Activity Questionnaire; MET, metabolic equivalent; PSV, primary systemic vasculitis

	IIM (n=132)	PSV (n=82)	CTR (n=178)	P-value	Statistic of test	P-value (post hoc)			Statistic of test (post hoc)		
						IIM vs. CTR	PSV vs. CTR	IIM vs. PSV	IIM vs. CTR	PSV vs. CTR	IIM vs. PSV
Comorbidities											
Systemic arterial hypertension	53 (40.1)	39 (47.6)	54 (30.5)	0.022	7.637‡	0.100	0.011	0.356	2.694‡	6.359‡	0.850‡
Diabetes mellitus	17 (12.9)	6 (7.3)	14 (7.9)	0.254	2.737‡	0.212	>0.999	0.293	1.554‡	<0.001‡	1.102‡
Dyslipidemia	24 (18.1)	17 (20.7)	31 (17.5)	0.821	0.393‡	0.998	0.654	0.777	<0.001‡	0.200‡	0.079‡
IPAQ											
Low	56 (42.4)	38 (46.3)	83 (46.6)	0.741	0.600‡	0.534	>0.999	0.675	0.385‡	<0.001‡	0.176‡
Moderate	41 (31.1)	24 (29.3)	34 (19.1)	0.036	6.627‡	0.021	0.095	0.901	5.277‡	2.787‡	0.015‡
High	35 (26.5)	20 (24.4)	61 (34.3)	0.171	3.534‡	0.182	0.146	0.853	1.784‡	2.115‡	0.034‡
METs (week)	897 (198-3600)	822 (260-2000)	2700 (446-5040)	<0.001	15.069††	<0.001	<0.001	0.601	4.053†††	4.902†††	1.342†††
Fatigue											
FSS (9.0-63.0)	38 (23.2-53.0)	35 (18.2-45.0)	26 (15.0-41.0)	<0.001	20.468††	<0.001	0.059	0.229	6.321†††	3.219†††	2.120†††
Quality of life											
EQ-5D (0.00-1.00)	0.69 (0.51-0.80)	0.73 (0.51-0.78)	0.78 (0.69-1.0)	<0.001	23.168††	<0.001	<0.001	0.989	6.055†††	6.055†††	0.196†††

The FSS score was higher in the IIM group than in the CTR and tended to be higher in the PSV group. The EQ-5D scores were lower in the IIM and PSV groups than in the CTR.

The groups were compared in terms of internet access, usage, and proficiency with cell phones and wearable devices. However, the knowledge and usage of wearable devices and smartphones did not exhibit a significant difference between the groups (Table 3).

**Table 3:** Use of smartphones and wearable devices Data are presented as the median (25th-75th) or number (%) *5 (a lot of skill); ‡chi-squared statistic CTR, control group; IIM, idiopathic inflammatory myopathies; PSV, primary systemic vasculitis

	IIM (n=132)	PSV (n=82)	CTR (n=178)	P-value	Statistic of test	P-value (post hoc)			Statistic of test (post hoc)		
						IIM vs. CTR	PSV vs. CTR	IIM vs. PSV	IIM vs. CTR	PSV vs. CTR	IIM vs. PSV
Use smartphone	128 (97.0)	81 (98.8)	177 (99.4)	0.299	3.131 ‡	0.211	>0.999	0.699	1.563‡	<0.001‡	0.149‡
Internet in smartphone	122 (95.3)	81 (98.8)	171 (96.6)	0.396	1.852‡	0.781	0.555	0.331	0.076‡	0.348‡	0.944‡
Ability to use a smartphone*	47 (35.6)	36 (43.9)	63 (35.6)	0.374	1.967‡	>0.999	0.239	0.286	<0.001‡	1.382‡	1.137‡
Know about wearable monitoring devices	104 (78.8)	65 (79.3)	144 (80.9)	0.891	0.231‡	0.752	0.888	>0.999	0.099‡	0.019‡	<0.001‡
Ever used wearable devices	30 (28.8)	20 (24.4)	47 (26.4)	0.757	0.557‡	0.543	0.847	0.909	0.369‡	0.037‡	0.013‡
Ability to use wearable devices*	12 (9.09)	12 (14.6)	15 (8.43)	0.276	2.578	0.998	0.191	0.305	<0.001‡	1705‡	1.053‡

The median age of the patients and volunteers who did not use wearable devices was 50 (41.0-58.0) years, whereas that of the patients and volunteers who used wearable devices was 38 (31.0-48.0) years.

**Figure 1:**
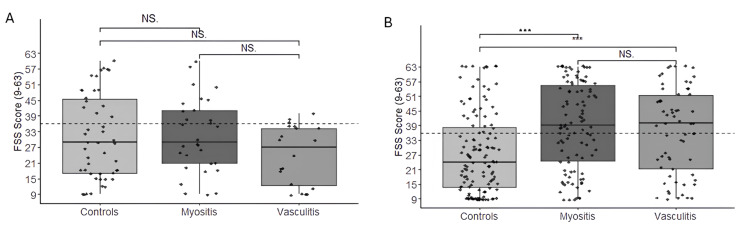
Severity score level of fatigue in patients and the CTR who used wearable devices (A) and those who did not use wearable devices (B) ***P < 0.001 CTR, control group; FSS, fatigue severity scale; NS, not significant

**Figure 2:**
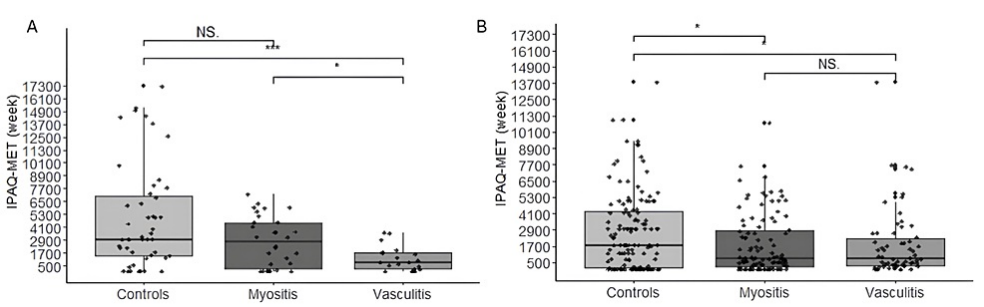
Physical activity level in patients and the CTR who used wearable devices (A) and those who did not use wearable devices (B) *P < 0.05; ***P < 0.001 CTR, control group; IPAQ, International Physical Activity Questionnaire; MET, metabolic equivalent; NS, not significant

As an additional analysis, we evaluated the levels of fatigue, physical activity, and quality of life among individuals who did and did not use wearable devices. Patients who used these devices scored 29 (18-40) points on the FSS (Figure 1A). Conversely, patients who did not use them had a higher score of 33 (17-49) points (Figure 1B) (P=0.0003; W=5462.0). Furthermore, the IPAQ-Met score (weeks) was higher among those who used the devices, indicating a higher level of physical activity compared to those who did not, as illustrated in Figures 2A, 2B (P=0.008; W=11757.0), respectively. However, when comparing the EQ-5D scores among the groups, no significant difference was observed among those who used the wearable devices.

## Discussion

In this study, we examined the use of low-complexity devices among patients with IIM and PSV groups compared to the CTR. Despite the low adoption rates of wearables, the patients were aware of the technology. Age was identified as a significant factor in device adoption, with younger patients more likely to use it. These devices may affect patients' quality of life, as evidenced by reduced fatigue and increased physical activity among users.

A positive aspect of the present study is the sampling of patients from rare groups of systemic autoimmune rheumatic diseases, the presence of a CTR for comparison of findings, and the opportunity to educate patients about wearable technology for implementation in daily life, especially for those who are unfamiliar with its multiple health-monitoring functions.

Most individuals in developed and developing societies use smartphones, as emphasized by Dixon et al. [19]. Additionally, approximately one-fifth of adults in the USA own wearable technologies. Our study revealed that although most patients and the CTR are familiar with smartphones and wearables, only one-third effectively use wearable technology. This suggests the limited adoption of wearables despite the patients’ and CTR group’s broad familiarity with them.

Germini et al. [9] demonstrated that the accessibility or acceptance of these tracking devices depends on the type of user or usage context. In other words, there are factors related to adoption, such as purpose of use, socioeconomic factors, and accuracy of the collected data, which may interfere with access to technology.

Furthermore, the factor that demonstrated an influence on device usage in this study was age. We observed that younger patients exhibited higher usage of wearable devices, as indicated by the median age. This aligns with the study by Moore et al. [20], who discussed how certain age-related characteristics can impact elderly individuals’ comfort with technology, such as low vision, limited dexterity, and technical skills.

Additionally, patients who used wearable devices showed lower levels of fatigue as indicated by the FSS score. As demonstrated in a study by Sandıkçı et al. [21], fatigue is a common and often persistent occurrence in rheumatic diseases and can have a significant impact on health-related quality of life.

Dos Santos et al. [22] emphasized that fatigue is also a commonly reported symptom among patients with PSV, which is considered a subtype of rheumatic disease. Managing fatigue through patient education regarding regular exercise and lifestyle changes can improve various symptoms, physical disability, and quality of life. Reinforcing this point, patients with IIM and PSV groups, when compared to the CTR, demonstrated higher FSS scores and lower levels of physical activity among those who did not use wearable devices.

The connection among wearable device use, lower FSS scores, and improved IPAQ-Met scores indicates their significant role in managing fatigue and encouraging an active lifestyle among patients. Conversely, An et al. [23] emphasized the health benefits of physical activity, including improved body composition, enhanced functional capacity, and a better lifestyle.

However, a limitation of this study is the absence of information regarding whether patients and the CTR were already engaging in physical activity before using wearables or if they initiated activity after adopting the technology. Thus, understanding the usage purposes, patients’ capability of self-management, and data accuracy is crucial for fully comprehending the benefits of this tool.

Ferguson et al. [24] demonstrated that the use of wearable devices is correlated with better physiological outcomes and improved quality of life, potentially attributable to increased physical activity. However, as reflected in our EQ-5D scores when comparing the groups, no significant difference was observed among those who used wearable devices.

Although there were no significant differences in EQ-5D scores, wearable devices could serve as effective complementary tools for lifestyle modifications and physical activity monitoring. Further research, especially targeting the elderly, is imperative to assess the factors influencing device use among patients, the prevalence of fatigue, and their knowledge of these devices to achieve more conclusive results and extended benefits for these patients.

The present study has some limitations. We analyzed our samples as IIM or PSV. Each is composed of different subtypes of systemic rheumatic autoimmune diseases (dermatomyositis, polymyositis, Takayasu arteritis, granulomatosis with polyangiitis, etc.). Second, we did not analyze disease activity or the current treatment of the patients, which may have interfered with our results. Third, no additional analyses (e.g., multivariate analysis) were performed because of the limited sample size.

## Conclusions

The use of wearable devices has the potential to enhance the quality of life of patients, and age is emerging as a significant factor in their adoption. However, only a small proportion of patients currently use these devices. Thus, facilitating the use of this technology may prove to be a valuable strategy for optimizing clinical treatment, particularly for the elderly population.
